# Interleukin-37b Suppressed ILC2s in Children with Allergic Rhinitis

**DOI:** 10.1155/2023/1572891

**Published:** 2023-04-14

**Authors:** Qingxiang Zeng, Yinhui Zeng, Haiqing Xiao, Jinyuan Li, Chao Yang, Renzhong Luo, Wenlong Liu, Yanhui Wen

**Affiliations:** ^1^Department of Otolaryngology, Guangzhou Women and Children's Medical Center, Guangzhou Medical University, Guangdong Provincial Clinical Research Center for Child Health, Guangzhou 510623, China; ^2^Department of Otolaryngology, Liuzhou hospital of Guangzhou Women and Children's Medical Center, Guangxi 545001, China; ^3^The Third People's Hospital of Dongguan, Dongguan Songshan Lake Center Hospital, Dongguan, China

## Abstract

**Background:**

Interleukin-37b is a fundamental inhibitor of innate and acquired immunity. Type 2 innate lymphoid cells (ILC2s) can secret type 2 cytokines and regulate allergic rhinitis (AR). However, the role of IL-37b in ILC2s in children with AR was not clear.

**Methods:**

We recruited 15 AR children and controls. The serum IL-37b levels and its relation with the frequency and functional phenotype of ILC2s. The regulation of IL-37b on ILC2s proliferation and function was confirmed using flow cytometry and enzyme-linked immunosorbent assay (ELISA). The mRNA expression of IL-1R8, IL-18R*α*, and ICOSL was examined using RCR. The change of IL-37b protein level in serum during subcutaneous allergen immunotherapy (SCIT) was determined by ELISA.

**Results:**

We have demonstrated that both of the frequencies of blood ILC2s, IL-5+ILC2s, and IL-13+ILC2s in AR children were elevated compared with controls. The serum protein level of IL-37b was downregulated in AR, and it was negatively related to the frequency of ILC2s, IL-5+ILC2s, and IL-13+ILC2s. IL-37b increased the mRNA levels of IL-1R8, IL-18R*α*, and ICOSL expressed by ILC2s. IL-37b suppressed the proliferation of ILC2s and the secretion of IL-5 and IL-13 from ILC2s. Finally, we found that IL-37b was increased in AR children after 3 years' SLIT, especially in the good response group.

**Conclusion:**

Our findings highlight the role of IL-37b in the suppression of ILC2s and establish a new therapeutic target in AR.

## 1. Introduction

Allergic rhinitis (AR) is a common disease that can be found in 15-25% of the population worldwide. AR often occurs in childhood, and the occurrence increases with increasing age [1]. The prevalence rate of children and adolescents was reported to be 44.23% [2]. The high incidence of AR has a significant financial impact on the management of the disease both direct and indirect. Allergen immunotherapy (AIT), mechanism-oriented therapy, is increasingly recognized as a most valuable therapy for AR patients, especially in the early phases [3, 4].

Type 2 innate lymphoid cells (ILC2) are innate immune cells that can secrete type II cytokines dependent on epithelium-derived cytokines IL-33, IL-25, and thymic stromal lymphopoietin (TSLP) [5]. The frequency of blood ILC2s was increased in AR patients and correlated with disease severity [6]. ILC2s can also express inducible T cell costimulator ligand (ICOSL), which mediated the interaction between ILC2s CD4+ T helper cell subsets [7–9].

IL-37, a member of the IL-1 family, can inhibit both natural and acquired immunity. IL-37 has five different isoforms, and IL-37b is the most important functional type [10]. IL-37b is detectable in human tissues including the liver, lung, bone marrow, and lymph nodes and can inhibit DCs functions as well as attenuate T cell-mediated inflammation [11]. IL-37b plays its role both intracellularly and extracellularly [12]. Intracellularly, IL-37b was cleaved and activated by caspase-1 and formed mothers against decapentaplegic homolog 3 (Smad3) in the nucleus. Extracellularly, IL-37b transduces anti-inflammatory signals by forming IL-37/IL1R8/IL-18R*α* complex [13–15]. Several studies demonstrated that IL-37b could downregulate IL-17 and IL-4 secretion by CD4+ T cells through interacting with IL1R8 in AR [16, 17].

We previously found that IL-37b had an anti-inflammatory effect in children with AR through downregulating with Th2 cytokines expressed by peripheral blood mononuclear cells [18]. Studies have also found IL-37b alleviates allergic symptoms by inhibiting Th2 and Th17 inflammation and suppressing the levels of IgE [13]. However, immunological roles and underlying mechanisms of IL-37b to ILC2s have remained elusive in allergic settings, especially in children with AR. Herein, it is worthy to explore the suppression role of IL-37b in ILC2s in AR children.

## 2. Methods

### 2.1. Subjects

All 15 AR children were recruited and confirmed according to disease history, physical examination, and positive skin prick test (Table 1). The 15 healthy controls matched age and gender. Subjects with severe systematic diseases and drug use history (oral or nasal corticosteroids, antiepileptics, and immunosuppressants) one month before the study were not included. This study received approval from the local Ethics Committee and the children's guardian.

### 2.2. Samples Collection and Cell Culture

The serum samples were achieved through 15 minutes centrifugation of 10 ml venous blood at 1000 × g. Ficoll density gradient was used for the purification of peripheral blood mononuclear cells (PBMC) from the blood of AR and controls.

Purified PBMCs (10^6^/mL) from AR patients were activated by phorbol myristate acetate (PMA, Sigma-Aldrich), ionomycin (Sigma-Aldrich), and Brefeldin A (Sigma-Aldrich) for 3 hours. Then, the ILC2s were sorted through staining by FITC lineage-negative cocktail kit, CD45, CRTH2, and CD127 (BD Bioscience). In detail, the lineage negative (Lin^−^) cells were obtained using the FITC lineage cocktail (CD2, CD3, CD14, CD16, CD19, CD56, and CD235a; BD Bioscience) and FceRI (eBioscience). Then, the Lin^−^ cells were stained with CD45, CRTH2, and CD127 (BD Bioscience) for isolation of ILC2s. Finally, the ILC2 was sorted using a FACS ARIA flow cytometer (BD Biosciences) with a purity of greater than 93%. Then, the data were analyzed by FlowJo software (TreeStar).

Sorted ILC2s (Lin^−^ CD45^+^CRTH2^+^CD127^+^) were incubated in 96-well plates with a density of 2.5 × 10^5^ cells/mL. The culture medium contained recombinant IL-25, IL-33, TSLP (all 10 ng/mL), IL-2 (50 ng/mL), or rhIL-37b (1–100 ng/mL) (all from the R&D Systems) for 72 hours. The ILC2s' proliferation was detected using the incorporation of tritiated thymidine.

### 2.3. Enzyme-Linked Immunosorbent Assay (ELISA)

The levels of cytokines from plasma or cell culture supernatant were examined by ELISA kits (R&D Systems). The detection limits were shown as follows: IL-5, 3.9 pg/mL; IL-13, 125 pg/mL; and IL-37b, 125 pg/mL.

### 2.4. The mRNA Level Detected by qRT-PCR

The total RNA from ILC2s was isolated using RNeasy Mini Kit. The reverse transcription of RNA (1 *μ*g) was done by cDNA kit (Qiagen). The cDNA synthesis was performed using an Oligo Primer and SuperScript III Reverse Transcriptase (Invitrogen). The target gene expression was calculated by the formula 2^−*ΔΔ*Ct^ and normalized to the endogenous gene [19].

### 2.5. Allergen Immunotherapy

Standardized allergen extracts (50% of *Dermatophagoides pteronyssinus* and 50% of *Dermatophagoides farinae*) (Allergopharma) were administered as described by the manufacturer's instruction. Briefly, at the initial buildup stage, the subjects received injections weekly from doses of 0.2 in no. 1 vials to 1.0 mL in the no. 3 vial. At the maintenance stage, 1.0 mL in the no. 3 vial was administered at 4-6 weeks interval. The efficacy of SCIT was determined using symptom medication score (SMS), which is the sum of total nasal symptom score (TNSS) and rescue medication score (RMS). The TNSS was scored from 0 to 3 (no, slight, moderate, and severe symptoms) according to severity. The RMS was scored according to medication use (1 for an oral or intranasal antihistamine and 2 for an intranasal corticosteroid) [20]. The evaluation and serum collection were performed at baseline and 3 years after SCIT. Children who obtained > 50% reduction of SMS 3 years after SCIT were defined good responses, while the others were defined as having poor responses.

### 2.6. Statistical Analysis

The data were provided as the mean ± standard deviation or medium and interquartile interval and analyzed by nonparametric Mann–Whitney U test.

Intergroup comparisons were performed followed by the post hoc Tukey's test. The Spearman correlation analysis was performed t. Statistical significant difference was set as 0.05.

## 3. Results

### 3.1. Comparisons of the Frequency and Functional Phenotype of ILC2 between AR and Control Children

**Figure 1:**
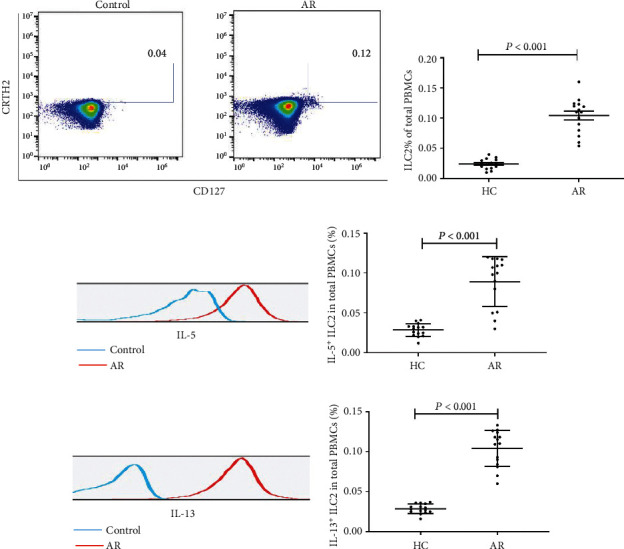
The frequency of ILC2, IL-5+ILC2, and IL-13+ILC2 cells between AR patients and controls. (a, c, e) Flow cytometric results of the proportion of ILC2, IL-5+ILC2, and IL-13+ILC2 cells between HC and AR. (b, d, f). The proportions of ILC2, IL-5+ILC2, and IL-13+ILC2 between HC and AR were detected by flow cytometry. AR: allergic rhinitis; HC: healthy control; ILC2: type 2 innate lymphoid cells.

Despite heterogeneity across donors, the frequency of ILC2s was higher in AR than in controls (Figure 1). As type 2 cytokines are associated with the pathophysiology of AR, we studied subsets of ILC2s and confirmed that the percentages of IL-5^+^ILC2s and IL-13^+^ILC2s were increased in the AR group than controls (Figure 1).

### 3.2. IL-37b Levels and Its Correlation with ILC2s and Type 2 Cytokines in AR

**Figure 2:**
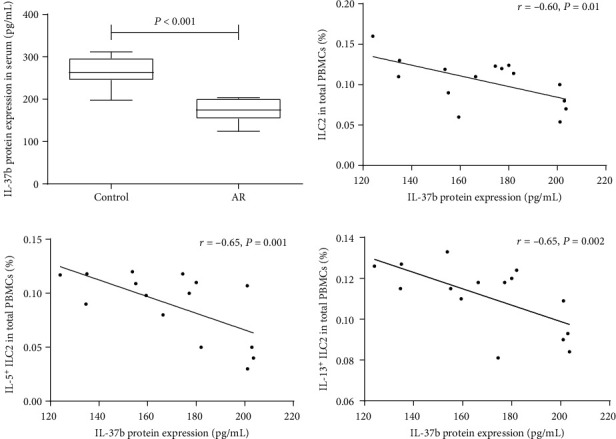
Correlation between IL-37b protein expression and the proportion of ILC2, IL-5+ILC2, and IL-13+ILC2 cells in AR. (a) The IL-37b protein expression between control and AR. (b–d) Negative correlation between IL-37b protein expression and the proportions of ILC2, IL-5+ILC2, and IL-13+ILC2 in AR. AR: allergic rhinitis; ILC2: type 2 innate lymphoid cells.

We next assessed the levels of IL-37b in serum from participants. Reduction of IL-37b in individuals with AR was found when compared with normal controls (Figure 2). We observed that the serum level of IL-37b was strongly negatively related to the frequency of ILC2s, IL-5^+^ILC2s, and IL-13^+^ILC2s (Figure 2), suggesting a potential suppression effect of IL-37b in type 2 immune response.

### 3.3. IL-37b Suppressed the Proliferation and Function of ILC2s

**Figure 3:**
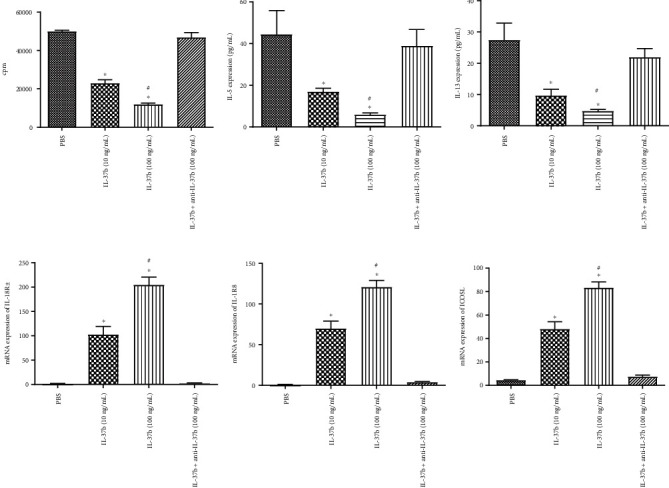
The ILC2 cell proliferation and cytokine expression regulated by IL-37b in AR patients. (a) The proliferation of ILC2 was determined using tritiated thymidine incorporation under IL-37b stimulation. (b, c) The expression of IL-5 and IL-13 by ILC2 under IL-37b stimulation was assessed by ELISA. (d–f) The mRNA expression of IL-1R8, IL-18R*α*, and ICOSL by ILC2 after stimulated by IL-37b. Three independent tests were performed for every experiment. ^∗^ILC2: type 2 innate lymphoid cells; ICOSL: inducible T cell costimulator ligand. Compared with the PBS group, *P* < 0.05; ^#^Compared with the IL-37b (10 ng/mL) group, *P* < 0.05.

As expected, the proliferation of ILC2s treated with IL-37b was suppressed and the production of IL-5 and IL-13 was clearly reduced (Figure 3). We also detected enhanced expression of IL-1R8, IL-18R*α*, and ICOSL in ILC2s (Figure 3) in a dose-dependent manner.

### 3.4. IL-37b Increased in AR Children after 3 Years' SCIT

**Figure 4:**
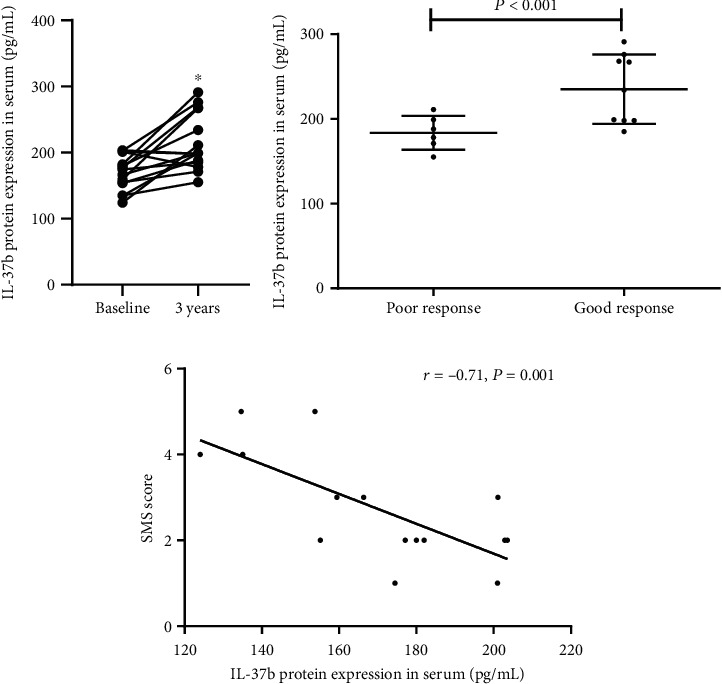
The protein expression of IL-37b during subcutaneous allergen immunotherapy. (a) The comparison of IL-37b protein expression between baseline and 3 years after SCIT. (b) The comparison of IL-37b protein expression between poor and good response groups 3 years after SCIT. (c) The correlation between IL-37b protein expression and disease severity. SMS: symptom medication score; SCIT: subcutaneous allergen immunotherapy. ^∗^Compared with the baseline group, *P* < 0.05.

The TNSS, RMS, and SMS after 3 years' SCIT decreased compared with the baseline (Table 2) in agreement with previous reports [20]. As to the serum level of IL-37b, we found an increase following the 3-year SCIT treatment, especially in the good response group (Figure 4). Moreover, the IL-37b expression and SMS score were negatively correlated after 3-year SCIT treatment (Figure 4).

## 4. Discussion

ILC2s play specific roles in promoting the development of AR. We found that the proliferation of ILC2s and their functional phenotype were increased in AR children. IL-37 functions as a suppressor in both innate and adaptive immune responses. Here, we confirmed that IL-37b was decreased in AR, and it can function as a critical inhibitor of ILC2s cells and the secretion of IL-5 and IL-13. Moreover, the study revealed that IL-37b participated in the pathogenesis of SCIT in children with AR via suppressing of ILC2s.

Allergens such as house dust mites can activate pattern recognition receptors in nasal epithelium to start innate immune responses by releasing alarmins (IL-33, TSPL, or IL-25) which could activate ILC2s to rapidly produce type 2 cytokine. These indicated that ILC2s have an important role in maintaining type 2 adaptive immune response [21]. Thus, we first confirmed the enhancement of ILC2s and functional phenotype in children with AR.

IL-37 can be secreted from diverse cells such as PBMCs and dendritic cells [11]. We inspected that the levels of IL-37b in children with AR were downregulated which is consistent with the previous reports [17, 18]. Our *in vitro* study found that IL-37b can regulate the proliferation and function of ILC2s negatively and directly. Consistently, rIL-37b treatment could reduce type 2 cytokines expression in the BALF of asthmatic mice [22].

IL-37 plays its role by binding to IL-18R*α* and IL-1R8 extracellularly [13]. We found that IL-37b could increase the levels of not only IL-18R*α* and IL-1R8 but also ICOSL in ILC2s, implying that IL-37b may regulate the interaction between ILC2 and Th2 cells.

For the clinical scenario, our data detected that the protein level of IL-37b was significantly upregulated in HDM-sensitive AR children after 3-year treatment with SCIT, which suggests that IL-37b might be involved in allergen-specific immunotherapy mediated by ILC2s. Our data also suggested that IL-37b levels were negatively correlated with disease severity, implying that IL-37b may be used as a biomarker during allergen-specific immunotherapy.

In summary, we revealed the suppression effects of IL-37b in children with AR through ILC2s. Our findings also highlight the potential of the treatment target of IL-37b/ILC2s.

## Figures and Tables

**Table 1:** Characteristics of subjects.

Characteristics	AR	Control
Cases	15	15
Age (years)	12.7 ± 4.5	13.6 ± 4.2
Male/female	8/7	7/8
Duration of symptoms (years)	2.3 ± 1.5	—
Positive to Der p	13	—
Positive to Der f	10	
Positive to cockroach	1	—
Positive to cat dander	1	—
Positive to mold	0	—
Positive to dog dander	1	—
TNSS	8.2 ± 3.5	—
RMS	2.7 ± 1.2	—
SMS	10.9 ± 4.6	—

Der p: *Dermatophagoides pteronyssinus*; Der f: *Dermatophagoides farinae*; TNSS: total nasal symptom score; RMS: rescue medication score; SMS: symptom medication score.

**Table 2:** Clinical efficacy of allergen immunotherapy.

	TNSS	RMS	SMS
Baseline score	8.2 ± 3.5	2.7 ± 1.2	10.9 ± 4.6
End-point score	2.5 ± 1.5^∗^	0.7 ± 0.3^∗^	3.1 ± 1.9^∗^

TNSS: total nasal symptom score; RMS: rescue medication score; SMS: symptom medication score; ^∗^Compared with baseline score, *P* < 0.05.

## Data Availability

The data used to support the findings of this study are available from the corresponding author upon request.
